# Hepatitis B virus RNA and hepatitis B surface antigen kinetics predict treatment outcomes in children with chronic hepatitis B

**DOI:** 10.3389/fcimb.2026.1746541

**Published:** 2026-02-03

**Authors:** Xiaorong Peng, Yunan Chang, Jiaying Wu, Jing Zhu, Peng Hu, Hong Ren, Hongmei Xu, Ruiqiu Zhao, Tao Qin

**Affiliations:** 1 Department of Infectious Diseases, Children’s Hospital of Chongqing Medical University, National Clinical Research Center for Children and Adolescents' Health and Diseases, Ministry of Education Key Laboratory of Child Development and Disorders, Chongqing Key Laboratory of Child Rare Diseases in Infection and Immunity, Chongqing, China; 2 Key Laboratory of Molecular Biology for Infectious Diseases (Ministry of Education), Department of Infectious Diseases, Institute for Viral Hepatitis, The Second Affiliated Hospital, Chongqing Medical University, Chongqing, China

**Keywords:** chronic hepatitis B (CHB), nucleos(t)ide analogue, pediatric, pregenomic RNA (pgRNA), treatment outcome

## Abstract

**Background:**

Serum hepatitis B virus (HBV) pregenomic RNA (pgRNA) predicts antiviral response in adults with chronic hepatitis B (CHB); however, its prognostic performance against conventional biomarkers in children remains underexplored. This study aimed to characterize the kinetics of pgRNA and hepatitis B surface antigen (HBsAg) and evaluate their predictive value for treatment outcomes in children with CHB.

**Methods:**

Sixty-five hepatitis B e antigen (HBeAg)-positive children with CHB who received ≥ 96 weeks of nucleos(t)ide analogue (NA) therapy were included. Serum viral biomarkers were measured at baseline, weeks 12, 48, and 96. Treatment outcomes were HBeAg seroconversion and HBsAg loss (Defined as HBsAg <100 IU/mL or seroclearance).

**Results:**

Patients initiating treatment before age 7 years had a significantly higher cumulative incidence of HBeAg seroconversion (73.5% vs. 48.4%) and HBsAg loss (50.0% vs. 9.7%) compared to older children (*p* < 0.05). Week 12 HBV pgRNA decline was an independent predictor of HBeAg seroconversion (area under the curve [AUC] = 0.793, 95% confidence interval [CI]: 0.686–0.900), outperforming HBV DNA and HBsAg kinetics. For predicting HBsAg loss, the week 12 HBsAg decline was an independent predictor (AUC = 0.762, 95% CI: 0.632–0.893), and its integration with patient age further improved predictive accuracy (AUC = 0.879, 95% CI: 0.796–0.962).

**Conclusions:**

Early pgRNA kinetics more accurately predict HBeAg seroconversion, whereas HBsAg dynamics forecast HBsAg loss more effectively, particularly when combined with patient age. This complementary monitoring strategy provides a clinically applicable tool for optimizing personalized management in children with CHB.

## Introduction

Chronic hepatitis B virus (HBV) infection remains a major global health challenge, accounting for approximately 1.1 million deaths in 2022 due to complications such as liver cirrhosis and hepatocellular carcinoma (World Health Organization, 2024). The pediatric population represents a critical reservoir for this burden. In particular, perinatal and early childhood infections carry a high risk of chronicity, leading to an estimated 5.6 million children under 5 years of age living with hepatitis B surface antigen (HBsAg) positivity worldwide (Terrault et al., 2018; Polaris Observatory Collaborators, 2023; You et al., 2023). While current guidelines recommend antiviral therapy for children with evidence of active disease or advanced fibrosis (Terrault et al., 2018; You et al., 2023; European Association for the Study of the Liver, 2025), clinical decision-making is complicated by the profound immunological heterogeneity across childhood. Notably, accumulating evidence indicates that younger children achieve superior treatment outcomes, including higher rates of hepatitis B e antigen (HBeAg) seroconversion and HBsAg loss, compared to older children and adults (Zhu et al., 2019; Huang et al., 2023; Li et al., 2023; Zhang et al., 2024). This efficacy gradient underscores the promise of age-stratified therapeutic strategies while revealing a central challenge—the lack of reliable, non-invasive tools to predict treatment response, which are essential for optimizing personalized management in pediatric chronic hepatitis B (CHB).

Conventional biomarkers, particularly serum HBV DNA and HBsAg, remain cornerstones of CHB management but are limited by inherent weaknesses in monitoring viral transcriptional activity. Once HBV DNA declines below detection thresholds during antiviral therapy, it fails to reflect the residual transcriptional activity of intrahepatic covalently closed circular DNA (cccDNA), the persistent episomal template for viral replication (Xia and Guo, 2020; Mak et al., 2023a). Furthermore, HBsAg production can originate from chromosomally integrated viral DNA fragments, decoupling its kinetics from active cccDNA transcription (Mak et al., 2023a; Taddese et al., 2025). These limitations highlight the critical need for novel biomarkers capable of dynamically quantifying cccDNA activity to guide personalized treatment approaches, particularly in children undergoing long-term therapy.

HBV pregenomic RNA (pgRNA), a 3.5-kb transcript directly transcribed from cccDNA, has emerged as a promising surrogate biomarker. It demonstrates a strong correlation with intrahepatic cccDNA levels (r = 0.25–0.89) and exhibits dynamic changes in response to antiviral interventions (Wang et al., 2017; Deng et al., 2022; Tian et al., 2025). In adult cohorts, pgRNA kinetics have been established as robust predictors of key treatment endpoints, including HBeAg seroconversion and HBsAg loss (van Bömmel et al., 2015; Xia et al., 2021; Kosaka et al., 2025). However, critical knowledge gaps persist in pediatric populations. The immunological and virological landscape of CHB in children differs markedly from that in adults, which precludes the direct extrapolation of adult data. To date, only limited studies have explored the utility of serum pgRNA for predicting HBeAg seroconversion in children (Wu et al., 2021; Lai et al., 2024), and its comparative prognostic performance against conventional virological markers (HBV DNA and HBsAg) remains inadequately characterized. Moreover, its role in predicting HBsAg loss (Defined as HBsAg < 100 IU/mL or seroclearance) in children is virtually unexplored.

Therefore, this study aimed to characterize the kinetics of serum viral markers (pgRNA, HBsAg, and HBV DNA) and evaluate their predictive performance for treatment response in a well-defined cohort of HBeAg-positive children receiving nucleos(t)ide analogues (NA) therapy. We sought to determine whether early pgRNA kinetics provide superior prognostic value over traditional biomarkers for predicting two critical endpoints: HBeAg seroconversion and HBsAg loss.

## Methods

### Study design and patients

This retrospective cohort study enrolled treatment-naïve, HBeAg-positive children with CHB at the Children’s Hospital of Chongqing Medical University between January 2017 and December 2024. All children were born to HBsAg-positive mothers and received a complete hepatitis B vaccination series after birth according to national guidelines. The inclusion criteria were as follows: 1) age 1–17 years; 2) HBsAg positivity for ≥ 6 months; 3) HBeAg-positive and anti-HBe-negative; and 4) completion of at least 96 weeks of NA therapy. The exclusion criteria were: 1) coinfection with other hepatitis viruses (HAV, HCV, HDV, or HEV), cytomegalovirus, Epstein-Barr virus, or human immunodeficiency virus; 2) coexistence of other liver diseases, including autoimmune hepatitis, drug-induced liver injury, metabolic disorders, or other known liver diseases; 3) evidence of liver cirrhosis, hepatic decompensation, or hepatocellular carcinoma; 4) history of immunosuppressive therapy; or 5) previous antiviral treatment.

Four patients aged < 2 years initially received lamivudine (LAM; 4 mg/kg/day) and were switched to entecavir (ETV; 0.015 mg/kg/day, maximum 0.5 mg/day) upon reaching ≥ 2 years of age, consistent with guideline recommendations on ETV age eligibility and to mitigate the risk of LAM-associated resistance (Terrault et al., 2018; You et al., 2023; European Association for the Study of the Liver, 2025). The remaining 61 patients received ETV monotherapy. All patients underwent regular clinical and laboratory assessments every 3–6 months. This study was approved by the Ethics Committee of the Children’s Hospital of Chongqing Medical University (Approval No. 2025-036). Written informed consent was obtained from the parents or legal guardians of all participants.

### Outcomes

Treatment outcomes were assessed at week 96. The primary outcome was HBeAg seroconversion, defined as HBeAg loss accompanied by the development of anti-HBe antibodies. The secondary outcome was HBsAg loss, defined as either HBsAg seroclearance or HBsAg levels < 100 IU/mL (Mak et al., 2023b; European Association for the Study of the Liver, 2025).

### Laboratory assessments

Serum biochemical parameters were measured using routine automated analyzers. Serum HBV DNA was quantified by real-time polymerase chain reaction (PCR) assays (Sansure Biotech, Changsha, China) with detection limits of 400 IU/mL (at baseline, weeks 12 and 48) and 10 IU/mL (at week 96). Serum HBsAg and HBeAg were detected using chemiluminescence microparticle immunoassay (CMIA) kits (Abbott GmbH & Co. KG, Wiesbaden, Germany). The lower limit of detection (LLOD) for HBsAg was 0.05 IU/mL; HBeAg values ≥ 1 s/co (sample rate/cut off rate) were considered positive. Serum HBV pgRNA was measured using an RNA capture probe-based assay (Rendu Biotechnology, Shanghai, China, Cat. 20213400174) with an LLOD of 50 copies/mL and linear range of 10²–10^8^ copies/mL. Hepatitis B core-related antigen (HBcrAg) was quantified using chemiluminescence enzyme immunoassay (Fujirebio, Tokyo, Japan) with a linear range of 3.0–7.0 log U/mL. Samples exceeding the upper quantification limit were appropriately diluted and retested.

### HBV genotyping

HBV genotypes were determined by real-time fluorescence quantitative PCR (Daan Gene, Guangzhou, China) and confirmed by direct sequencing of the pre-S/S gene, with comparisons to NCBI GenBank reference sequences.

### Histological analysis

Ultrasound-guided percutaneous liver biopsies were performed in 62 patients at baseline. Liver tissue specimens were evaluated by senior pathologists blinded to clinical data. Histological grading of inflammation (G0–4) and staging of fibrosis (S0–4) were assessed according to Scheuer’s criteria. Intrahepatic cccDNA was quantified in 38 available liver samples using the QX200 droplet digital PCR system (Bio-Rad Laboratories, Hercules, California, USA), following the manufacturer’s protocol.

### Statistical analysis

Statistical analyses were performed using GraphPad Prism (version 10.0) and MedCalc (version 23.3.1). Viral biomarkers (HBV DNA, HBsAg, and pgRNA) were log_10_-transformed for analysis; measurements below the detection threshold were assigned a value equal to the respective LLOD. Continuous variables were expressed as median with interquartile ranges [Median (P25, P75)] and compared using Mann-Whitney U or Wilcoxon signed-rank tests. Categorical variables were presented as numbers (percentages) and compared using Chi-squared or Fisher’s exact tests, as appropriate. Kaplan-Meier survival analysis was performed to compare the cumulative incidence of HBeAg seroconversion and HBsAg loss between pre-defined age groups (with the log-rank test). Correlations between variables were assessed using Spearman’s rank correlation coefficient. To identify independent factors associated with treatment outcomes, multivariate logistic regression was performed using variables that were significant in univariate analysis (*p* < 0.05). The predictive performance of key variables was evaluated using receiver operating characteristic (ROC) curve analysis, and the DeLong test was used to compare the areas under the ROC curves (AUCs). A two-sided p-value < 0.05 was considered statistically significant.

## Results

### Baseline characteristics

**Figure 1:**
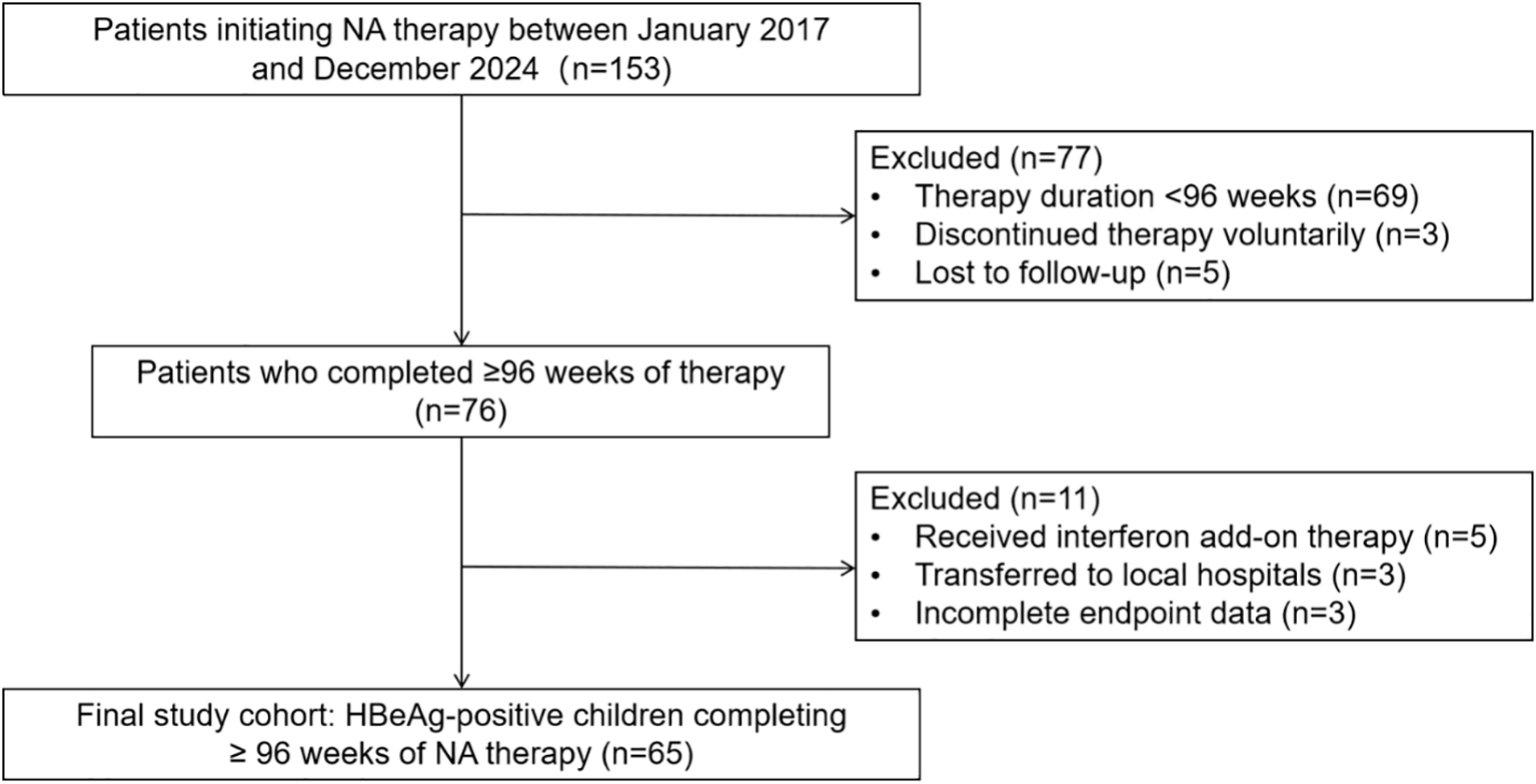
Flowchart of patient selection. NA, nucleos(t)ide analogue; HBeAg, hepatitis B e antigen.

A total of 65 patients (58.5% male) were enrolled for analysis (**Figure 1**). All children were born to HBsAg-positive mothers and received a complete hepatitis B vaccination series after birth according to national guidelines. The median age of the cohort was 6.84 (4.29–10.64) years, with a predominance of HBV genotype B. Median levels of HBV DNA, HBsAg, pgRNA, and HBcrAg were 8.03 (7.34–8.46) log_10_ IU/mL, 4.24 (3.77–4.67) log_10_ IU/mL, 7.27 (6.53–7.77) log_10_ copies/mL, and 8.30 (8.10–8.50) log_10_ U/mL, respectively. Histological assessment revealed moderate inflammation (G = 2) in 59.7% (37/62) and significant fibrosis (S ≥ 2) in 29.0% (18/62) of patients. The median intrahepatic cccDNA level (n = 38) was 12.00 (4.55–20.95) copies/cell (**Table 1**).

**Table 1:** Demographic and baseline characteristics (n=65).

Characteristics	Value
Male	38 (58.5)
Age, years	6.84 (4.29–10.64)
ALT, U/L	133.70 (71.80–292.50)
AST, U/L	95.00 (59.49–190.00)
HBV DNA, log_10_ IU/mL	8.03 (7.34–8.46)
HBsAg, log_10_ IU/mL	4.24 (3.77–4.67)
HBV pgRNA, log_10_ copies/mL	7.27 (6.53–7.77)
HBcrAg, log_10_ U/mL	8.30 (8.10–8.50)
Intrahepatic cccDNA^a^, copies/cell	12.00 (4.55–20.95)
Grade of inflammation^b^	
G1	25 (40.3)
G2	37 (59.7)
Stage of fibrosis^b^	
S0	4 (6.5)
S1	40 (64.5)
S2	15 (24.2)
S3	3 (4.8)
HBV genotype	
B	39 (60.0)
C	21 (32.3)
Undetected	5 (7.7)

Values are presented as numbers (%) or median (interquartile range).

ALT, alanine aminotransferase; AST, aspartate aminotransferase; HBV, hepatitis B virus; HBsAg, hepatitis B surface antigen; pgRNA, pregenomic RNA; HBcrAg, hepatitis B core-related antigen; cccDNA, covalently closed circular DNA.

^a^Data for intrahepatic cccDNA were available in 38 patients. ^b^Liver histological analyses were performed in 62/65 patients.

### Treatment outcomes

**Figure 2:**
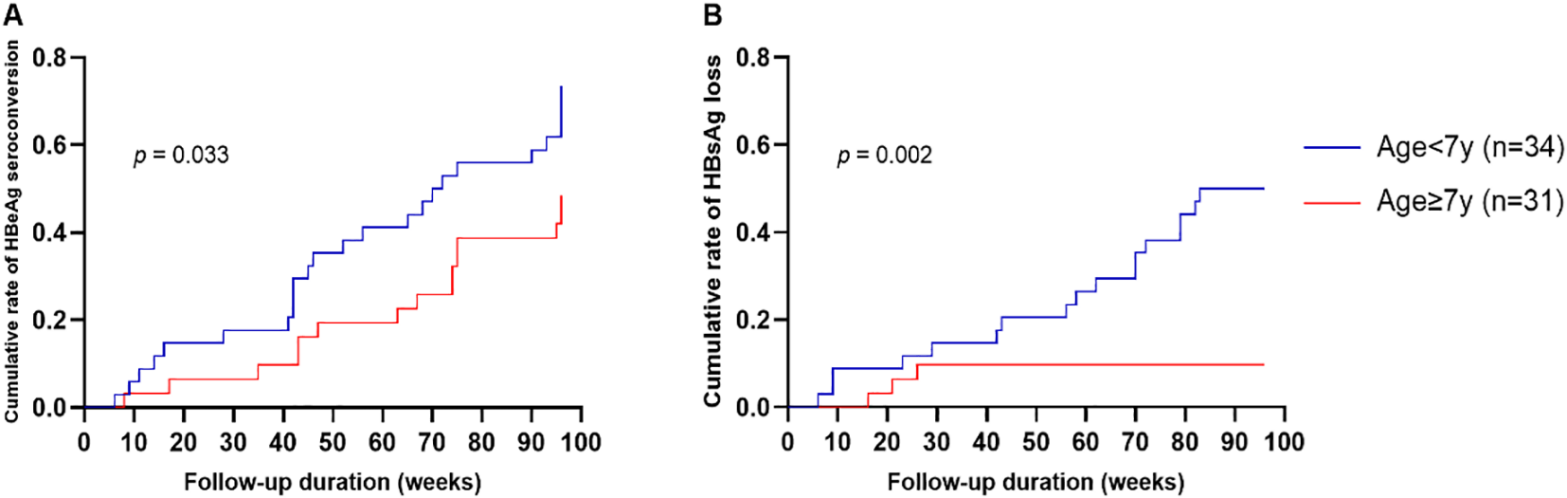
The cumulative incidence of HBeAg seroconversion and HBsAg loss in different age groups. Kaplan-Meier curves compare the cumulative incidence of HBeAg seroconversion **(A)** and HBsAg loss **(B)** between children who started antiviral therapy before and after 7 years of age. Statistical significance was determined using the log-rank test. HBeAg, hepatitis B e antigen.

After 96 weeks of NA therapy, the cumulative incidence rates of HBeAg seroconversion and HBsAg loss were 61.5% (40/65) and 30.8% (20/65), respectively. Logarithmic rank test showed that, compared with older children, patients who started treatment before the age of 7 had higher cumulative incidence of HBeAg seroconversion (73.5% vs. 48.4%, *p* = 0.033, **Figure 2A**) and HBsAg loss (50.0% vs. 9.7%, *p* = 0.002, **Figure 2B**). A complete HBeAg seroconversion rate (100%) was observed in the 1–3-years subgroup (n = 6), although this finding requires cautious interpretation due to the small sample size.

### Viral biomarker kinetics during antiviral therapy

**Figure 3:**
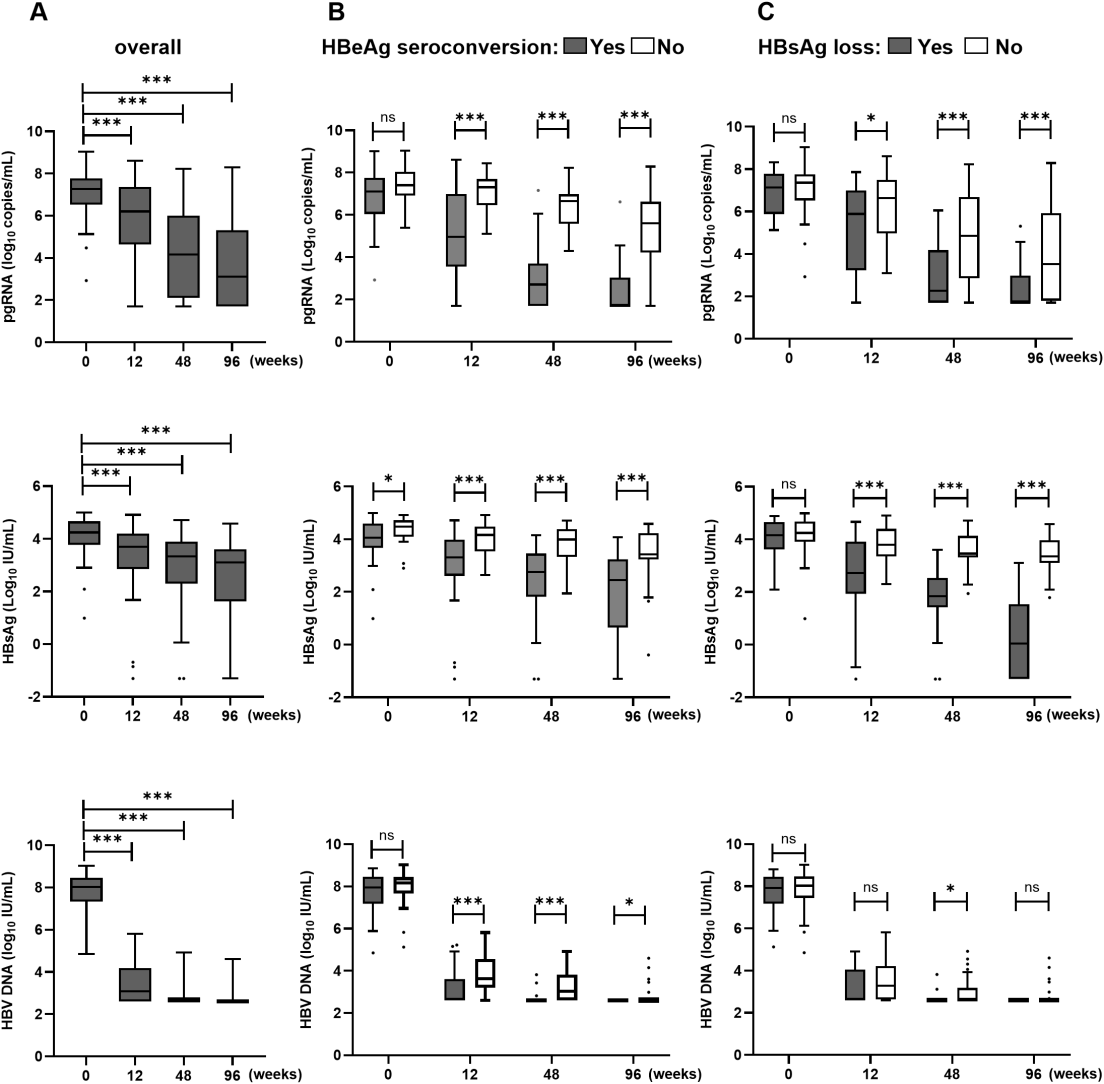
Summary of HBV biomarker kinetics from baseline to 96 weeks post-treatment. Kinetics of serum HBV pgRNA, HBsAg, and HBV DNA levels are shown in all patients **(A)**, patients with or without HBeAg seroconversion **(B)**, and patients with or without HBsAg loss **(C)**. ns = non-significant (*p* > 0.05), *p < 0.05, **p < 0.01, ***p < 0.001. HBeAg, Hepatitis B e antigen; HBV, hepatitis B virus; HBsAg, hepatitis B surface antigen; pgRNA, pregenomic RNA. * indicates statistical significance, and presented in the figure.

Longitudinal monitoring revealed differential suppression kinetics among the viral biomarkers (**Figure 3A**). HBV DNA demonstrated rapid clearance, declining from 8.03 (7.34–8.46) log_10_ IU/mL at baseline to 3.08 (2.60–4.19) log_10_ IU/mL at week 12. By week 48, the median viral load had reached the assay’s LLOD (400 IU/mL). The proportion of patients with undetectable HBV DNA (< 400 IU/mL) increased progressively, from 32.3% at week 12 to 73.8% at week 48 and 90.8% at week 96. In contrast, HBV pgRNA exhibited a more gradual decline, from 7.27 (6.53–7.77) log_10_ copies/mL at baseline to 6.24 (4.65–7.37), 4.15 (2.12–6.00), and 3.10 (1.70–5.33) log_10_ copies/mL at weeks 12, 48, and 96, respectively. Corresponding undetectable pgRNA rates (< 50 copies/mL) rose progressively to 4.6%, 18.5%, and 35.4% at these time points (**Supplementary Figure 1**). Notably, pgRNA remained detectable in 59.2% (29/49) of patients who achieved profound HBV DNA suppression (< 10 IU/mL) by week 96. Serum HBsAg showed the most attenuated decline throughout the treatment period, decreasing from 4.24 (3.77–4.67) log_10_ IU/mL at baseline to 3.11 (1.47–3.60) log_10_ IU/mL at week 96.

### Correlations between serum pgRNA and other viral biomarkers

At baseline, serum HBV pgRNA levels correlated strongly with HBV DNA (r = 0.693, *p* < 0.001), HBsAg (r = 0.553, *p* < 0.001), and HBcrAg (r = 0.413, *p* < 0.001) (**Table 2**). In the subset of 38 patients with available data, intrahepatic cccDNA levels correlated significantly with serum pgRNA (r = 0.424, *p* = 0.008), HBV DNA (r = 0.505, *p* = 0.001), and HBcrAg (r = 0.331, *p* = 0.049), but not with HBsAg (r = 0.156, *p* = 0.348). No significant correlations were observed between baseline pgRNA levels and host factors including HBV genotype, age, sex, or alanine aminotransferase (ALT) levels (all *p* > 0.05). Longitudinal analyses revealed persistent correlations between pgRNA and both HBV DNA (r range = 0.474–0.800; *p* < 0.001) and HBsAg (r range = 0.553–0.661; *p* < 0.001) throughout the 96 weeks of therapy. However, the correlation coefficients exhibited a declining trend over time (**Supplementary Table 1**).

**Table 2:** Correlation coefficients between viral biomarkers at baseline (n=65).

HBV marker	HBV DNA	HBsAg	HBcrAg	cccDNA
HBV pgRNA	0.693 (*p* < 0.001)	0.553 (*p* < 0.001)	0.413 (*p* = 0.001)	0.424 (*p* = 0.008)
HBV DNA	–	0.588 (*p* < 0.001)	0.316 (*p* = 0.016)	0.505 (*p* = 0.001)
HBsAg	–	–	0.454 (*p* < 0.001)	0.156 (*p* = 0.348)
HBcrAg	–	–	–	0.331 (*p* = 0.049)

HBV, hepatitis B virus; HBsAg, hepatitis B surface antigen; pgRNA, pregenomic RNA; HBcrAg, hepatitis B core-related antigen; cccDNA, covalently closed circular DNA.

### Association of biomarker kinetics with treatment response

Patients who achieved HBeAg seroconversion or HBsAg loss exhibited significantly lower pgRNA and HBsAg levels at all timepoints compared to non-responders (all *p* < 0.05) (**Figures 3B, C**). The magnitude of decline from baseline for both biomarkers was also considerably greater in responders. Specifically, HBeAg seroconverters demonstrated larger declines in serum pgRNA at all intervals and in HBsAg from week 48 onward (p < 0.05). Patients attaining HBsAg loss showed greater reductions in both biomarkers throughout treatment compared with those who did not (all *p* < 0.05). In contrast, HBV DNA suppression was rapid and profound but did not differ significantly between response groups (*p* > 0.05) (**Supplementary Table 2**). The detailed statistical comparisons for these group differences are provided in **Supplementary Table 3** (for HBeAg seroconversion) and **Supplementary Table 4** (for HBsAg loss).

Multivariate logistic regression analysis identified younger age at treatment initiation as an independent predictor for both HBeAg seroconversion (odds ratio [OR] = 0.540, 95% confidence interval [CI]: 0.343–0.730; *p* < 0.001) and HBsAg loss (OR = 0.762, 95% CI: 0.589–0.928; *p* = 0.016). Elevated baseline ALT (OR = 1.018, 95% CI: 1.006–1.035; *p* = 0.015) and greater week 12 pgRNA decline (OR = 4.393, 95% CI: 1.383–21.680; *p* = 0.030) were additional independent predictors of HBeAg seroconversion. In contrast, greater week 12 HBsAg decline was an independent predictor of HBsAg loss (OR = 5.525, 95% CI: 1.720–25.010; *p* = 0.011) (**Table 3**).

**Table 3:** Logistic regression analysis for predictors of HBeAg seroconversion and HBsAg loss.

Variable	HBeAg seroconversion				HBsAg loss			
	Univariate analysis		Multivariate analysis		Univariate analysis		Multivariate analysis	
	OR (95% CI)	*p*-value	OR (95% CI)	*p*-value	OR (95%CI)	*p*-value	OR (95%CI)	*p*-value
Age, years	0.836(0.721-0.957)	0.013	0.540(0.343-0.730)	<0.001	0.810(0.672-0.945)	0.015	0.762(0.589-0.928)	0.016
Sex (male as reference)	1.179(0.425-3.255)	0.750			0.607(0.207-1.766)	0.358		
Baseline ALT, U/L	1.011(1.005-1.019)	0.002	1.018(1.006-1.035)	0.015	1.001(0.998-1.004)	0.389		
Baseline HBcrAg, log_10_ U/mL	0.575(0.173-1.310)	0.284			3.337(1.008-17.820)	0.103		
Baseline HBV pgRNA, log_10_ copies/mL	0.627(0.347-1.036)	0.090			0.898(0.554-1.471)	0.657		
Baseline HBsAg, log_10_ IU/mL	0.361(0.120-0.883)	0.023			0.802(0.378-1.726)	0.554		
Baseline HBV DNA, log_10_ IU/mL	0.746(0.398-1.296)	0.321			0.833(0.477-1.475)	0.516		
Week12 ΔHBV pgRNA, log_10_ copies/mL	2.956(1.709-6.028)	<0.001	4.393(1.383-21.680)	0.030	1.624(1.131-2.435)	0.012		
Week12 ΔHBsAg, log_10_ IU/mL	1.983(1.107-4.235)	0.042			3.370(1.745-7.792)	0.001	5.525(1.720-25.010)	0.011
Week12 ΔHBV DNA, log_10_ IU/mL	1.650(0.955-3.009)	0.083			1.088(0.619-1.937)	0.770		

HBeAg, hepatitis B e antigen; HBsAg, hepatitis B surface antigen; HBV, hepatitis B virus; ALT, alanine aminotransferase; pgRNA, pregenomic RNA; HBcrAg, hepatitis B core-related antigen; OR, Odds Ratio; CI, confidence interval.

### Predictive performance of biomarker kinetics

**Figure 4:**
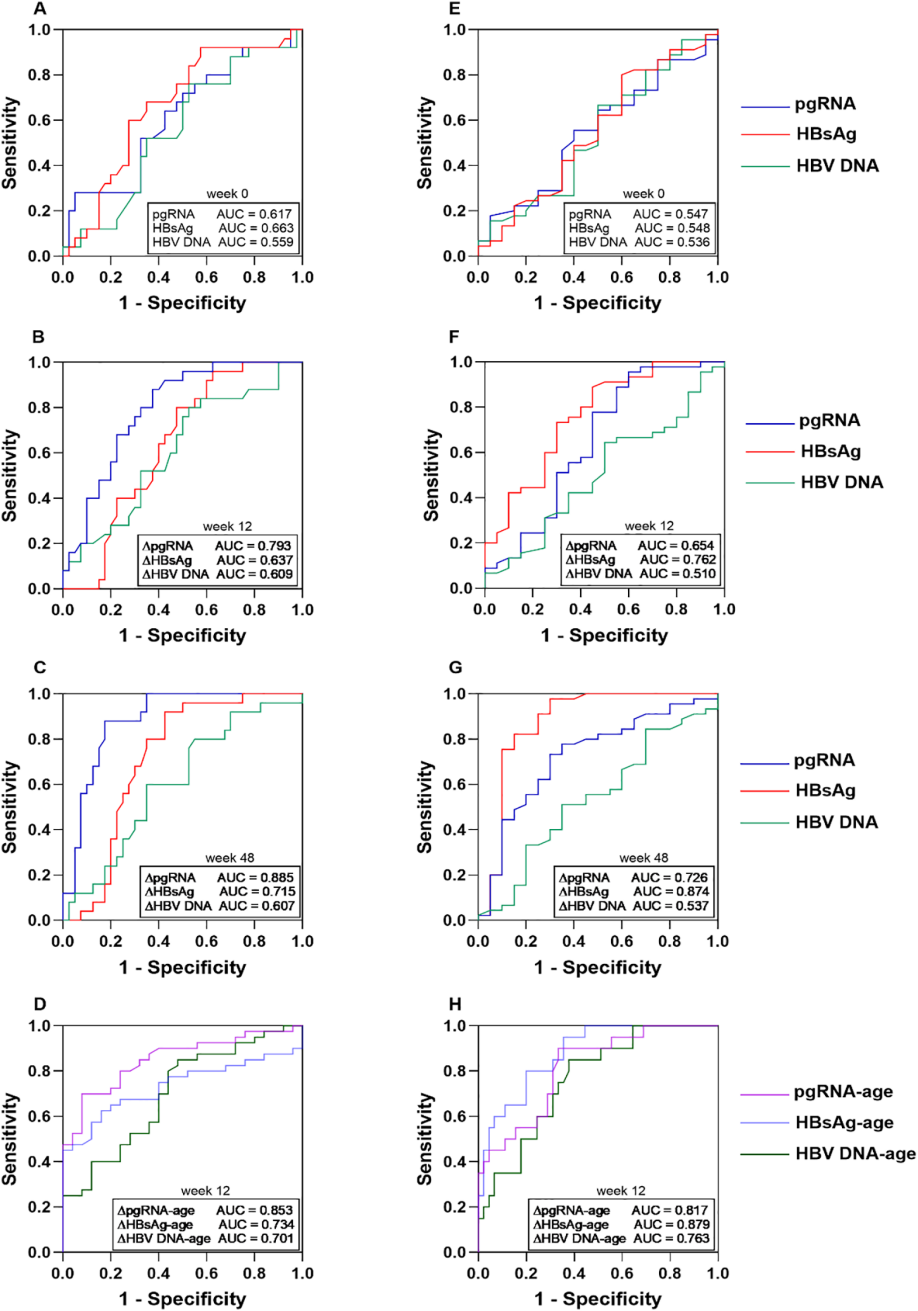
Predictive performance of viral biomarker kinetics and integrated models. Receiver operating characteristic (ROC) curves show the predictive power of baseline levels **(A)**, week 12 **(B)**, and week 48 **(C)** declines in viral biomarkers (HBV DNA, HBsAg, and pgRNA) for HBeAg seroconversion; baseline levels **(E)**, week 12 **(F)**, and week 48 **(G)** declines for HBsAg loss; and the integrated models combining age with week 12 kinetics for HBeAg seroconversion **(D)** and HBsAg loss **(H)**. HBeAg, Hepatitis B e antigen; HBsAg, hepatitis B surface antigen; pgRNA, pregenomic RNA.

ROC analysis revealed that baseline pgRNA and HBV DNA showed limited predictive value for both endpoints (all AUCs < 0.650, *p* > 0.05, **Figure 4A**), and baseline HBsAg offered only modest ability for predicting HBeAg seroconversion (AUC = 0.663, *p* = 0.028) (**Figure 4E**). In contrast, the kinetic changes of pgRNA and HBsAg during treatment demonstrated superior performance. For HBeAg seroconversion, the decline in pgRNA significantly outperformed the decline in HBsAg at both week 12 (AUC = 0.793 vs. 0.637; ΔAUC = 0.156, *p* = 0.022, **Figure 4B**) and week 48 (AUC = 0.885 vs. 0.715; ΔAUC = 0.170, *p* = 0.002) (**Figure 4C**). Using optimal thresholds (≥ 1.09 log_10_ copies/mL at week 12; ≥ 2.40 log_10_ copies/mL at week 48), pgRNA decline yielded a sensitivity/specificity of 62.5%/88.0% and 82.5%/88.0%, respectively. Consistent with its strong predictive value, patients stratified by the week 12 pgRNA decline cutoff (≥1.09 log_10_ copies/mL) showed a significantly higher and faster cumulative incidence of HBeAg seroconversion (Log-rank *p* < 0.001; **Supplementary Figure 2**).

For predicting HBsAg loss, a decline in HBsAg at week 12 was a strong predictor (AUC = 0.762, 95% CI: 0.632–0.893; *p* < 0.001), exhibiting a numerical advantage over pgRNA decline (AUC = 0.654; *p* = 0.10) (**Figure 4F**). By week 48, HBsAg decline achieved superior discrimination (AUC = 0.874, 95% CI: 0.754–0.993), significantly exceeding that of pgRNA decline (ΔAUC = 0.148, *p* = 0.002) (**Figure 4G**). HBV DNA decline showed no significant predictive value for either endpoint (all AUCs < 0.650, *p* > 0.05).

We further developed integrated prognostic models by combining the key host factor, age at treatment initiation (dichotomized at < 7 vs. ≥ 7 years), with viral biomarker kinetics. For predicting HBsAg loss, these integrated models achieved significantly higher predictive accuracy compared to biomarker kinetics alone (all *p* < 0.05 for ΔAUCs) (**Figure 4H**). Notably, the model combining age with week 12 HBsAg decline demonstrated the highest performance (AUC = 0.879, 95% CI: 0.796–0.962). In contrast, for HBeAg seroconversion, the combination of age with biomarker kinetics showed numerical but not statistically significant improvement over biomarker kinetics alone (all *p* > 0.05 for ΔAUCs) (**Figure 4D**).

## Discussion

This study establishes a clinically applicable prognostic framework for NA-treated pediatric CHB by defining the distinct and complementary predictive roles of early viral biomarker kinetics and a key host factor—patient age. We found that week 12 serum pgRNA decline was a robust and superior predictor of HBeAg seroconversion, outperforming HBV DNA and HBsAg kinetics. In contrast, early HBsAg reduction emerged as a strong predictor of HBsAg loss. Furthermore, younger age at treatment initiation was confirmed as a critical determinant of favorable treatment outcomes. Critically, the integration of age at treatment initiation with these biomarker kinetics markedly enhanced prognostic accuracy for HBsAg loss, offering a practical strategy for personalizing management in children with CHB.

Our results revealed a pronounced age-dependent therapeutic advantage. Children initiating NA therapy before age 7 achieved considerably higher cumulative rates of both HBeAg seroconversion and HBsAg loss than their older counterparts. Multivariate analysis further confirmed younger age as an independent predictor for both endpoints, reinforcing findings from prior studies. For instance, Zhang et al (Zhang et al., 2024). reported superior serological outcomes in children aged 1–7 years compared to those aged 7–16 years after 24 months of ETV-based therapy. Similarly, Wu et al (Wu et al., 2023). documented a strong inverse correlation between age and HBsAg clearance. The complete HBeAg seroconversion rate (100%) observed in our 1–3-year subgroup, despite its small sample size, further supports the potential efficacy of early intervention. Mechanistically, this enhanced treatment responsiveness in younger children likely stems from reduced cccDNA reservoirs, limited viral integration due to shorter infection duration, and preserved HBV-specific T-cell immunity (Kennedy et al., 2012; Le Bert et al., 2020; Wang et al., 2024). Collectively, these findings provide a strong rationale for considering earlier intervention in young, HBeAg-positive children with CHB.

Our data confirm a strong baseline correlation between serum pgRNA and intrahepatic cccDNA levels, establishing circulating pgRNA as a reliable surrogate for intrahepatic transcriptional activity in children with CHB. This validation addresses a crucial clinical need, given the impracticality of serial liver biopsies for monitoring cccDNA dynamics. Furthermore, the strong positive correlations between baseline serum pgRNA and other viral parameters (HBV DNA, HBsAg, and HBcrAg) are consistent with previous reports (Wu et al., 2021; Mak et al., 2023a; Lai et al., 2024; Taddese et al., 2025). This concordance reinforces the role of serum pgRNA as a reliable virological indicator across diverse age groups.

Longitudinal analysis revealed a progressive kinetic divergence between pgRNA and HBV DNA during NA therapy. The correlation coefficient declined from 0.800 at week 12 to 0.474 by week 96, a trend consistent with previously reported attenuation of pgRNA–DNA correlations under ETV treatment (Wu et al., 2021). While HBV DNA was rapidly cleared, pgRNA suppression occurred more gradually and remained detectable in over half of the children who had achieved profound virological suppression (HBV DNA <10 IU/mL) at week 96. The prevalence of pgRNA persistence (59.2%) is markedly lower than that reported in adults (77.5%) after a comparable NA therapy duration (Mak et al., 2021), suggesting a possible age-related advantage in transcriptional silencing. This kinetic dichotomy reflects differential antiviral mechanisms: NAs directly inhibit DNA synthesis, whereas pgRNA decline mirrors the progressive depletion and transcriptional silencing of the intrahepatic cccDNA reservoir (Fanning et al., 2019; Martinez et al., 2021; Mak et al., 2025). Therefore, persistent detection of pgRNA can serve as a clinically useful indicator of residual cccDNA activity, particularly in the presence of virological suppression.

As a direct transcriptional product of intrahepatic cccDNA, serum pgRNA is a robust predictor of HBeAg seroconversion. In our cohort, HBeAg seroconverters exhibited considerably greater reductions in pgRNA at all timepoints. The decline in pgRNA at both weeks 12 and 48 demonstrated good predictive accuracy for HBeAg seroconversion, markedly outperforming the declines in HBV DNA and HBsAg. Moreover, the week 12 pgRNA decline was identified as an early independent predictor of HBeAg seroconversion. These results are consistent with adult studies demonstrating the predictive superiority of pgRNA for HBeAg seroconversion (van Bömmel et al., 2015; Wu et al., 2025) and are further supported by emerging pediatric data. Previous studies reported strong predictive values for week 12/24 pgRNA levels and confirmed the predictive value of week 36 pgRNA decline during combination therapy (Wu et al., 2021; Lai et al., 2024). Collectively, the monitoring of serum pgRNA dynamics facilitates the early identification of treatment responders in children, providing a valuable tool to guide clinical decision-making.

In contrast, HBsAg demonstrated a more gradual decline during NA therapy. This attenuated response originates from its dual cellular origins: constitutive expression from chromosomally integrated HBV DNA fragments (unaffected by NA suppression) and ongoing transcription from residual cccDNA reservoirs (Mak et al., 2023a; Taddese et al., 2025). Consequently, HBsAg kinetics become increasingly dissociated from direct viral transcription markers. The magnitude of HBsAg reduction thus functions as a composite biomarker, reflecting both the clearance of viral antigens derived from integrated DNA and the extent of immune-mediated control over cccDNA. The persistent yet quantifiable decline in HBsAg renders it especially valuable for predicting HBsAg-related endpoints (Lim et al., 2021; Lee et al., 2024).

Achieving HBsAg levels < 100 IU/mL is strongly associated with a higher probability of functional cure (Ghany et al., 2023; Mak et al., 2023b; European Association for the Study of the Liver, 2025). In our cohort, the predictive power of HBsAg kinetics for this outcome strengthened over time. While week 12 HBsAg decline was an independent predictor, its discriminatory performance became notably superior to that of pgRNA by week 48. This pattern aligns with the biological premise that HBsAg kinetics better capture the complex process of immune-mediated control required for functional cure. Therefore, while pgRNA quantification more accurately reflects residual transcriptional activity, HBsAg monitoring remains essential for forecasting serological outcomes related to its own clearance.

A key innovation of our study lies in the development of integrated prognostic models that incorporate both viral and host factors. We demonstrated that combining patient age at treatment initiation with early kinetic changes in viral biomarkers considerably improved predictive accuracy for HBsAg loss compared to biomarker kinetics alone. Notably, the model integrating age with week 12 HBsAg decline exhibited the highest predictive performance (AUC = 0.879), offering a clinically applicable tool to identify children most likely to progress toward functional cure. The rationale for this approach is that functional cure necessitates both potent viral suppression and effective immune control—the latter being highly dependent on host immunological competence, which is strongly influenced by age in pediatric CHB. For the endpoint of HBeAg seroconversion, the integration of age with biomarker kinetics showed a numerical but not statistically significant improvement in predictive accuracy.

Our study has some limitations. First, its single-center, retrospective design carries an inherent risk of selection bias. The participants were primarily children from Southwest China with genotypes B or C, potentially limiting the generalizability to populations with other genotypes or ethnic backgrounds. Second, the sample size was relatively modest, particularly in the 1–3-year age subgroup, warranting caution in interpreting the corresponding results. Third, the higher HBV DNA detection threshold (400 IU/mL) at some timepoints precluded low-level viremia quantification, potentially obscuring more nuanced viral kinetic patterns. Finally, the 96-week follow-up period is insufficient to assess long-term outcomes such as sustained functional cure. Despite these limitations, our study provides compelling evidence for the complementary predictive value of pgRNA and HBsAg kinetics and pioneers a novel combined prognostic model in pediatric CHB. Future prospective, multi-center studies with larger cohorts and extended follow-up are warranted to validate these findings.

## Conclusions

Our findings propose a novel prognostic strategy for pediatric CHB by delineating the distinct and complementary roles of viral kinetics. The early decline in pgRNA is superior to traditional biomarkers in predicting HBeAg seroconversion, whereas early reduction in HBsAg effectively predicts HBsAg loss. Furthermore, initiating treatment at a younger age (particularly < 7 years) is strongly associated with improved outcomes. The integration of age with week 12 reductions in these biomarkers markedly enhanced the prediction of HBsAg loss, providing a clinically applicable tool for personalized management and early identification of treatment responders in children with CHB.

## Data Availability

The original contributions presented in the study are included in the article/**Supplementary Material**. Further inquiries can be directed to the corresponding author.

## References

[B1] Deng R. Liu S. Shen S. Guo H. Sun J. (2022). Circulating HBV RNA: From biology to clinical applications. Hepatology. 76, 1520–1530. doi: 10.1002/hep.32479, PMID: 35342969

[B2] European Association for the Study of the Liver (2025). EASL Clinical Practice Guidelines on the management of hepatitis B virus infection. J. Hepatol. 83, 502–583. doi: 10.1016/j.jhep.2025.03.018, PMID: 40348683

[B3] Fanning G. C. Zoulim F. Hou J. Bertoletti A. (2019). Therapeutic strategies for hepatitis B virus infection: towards a cure. Nat. Rev. Drug Discov. 18, 827–844. doi: 10.1038/s41573-019-0037-0, PMID: 31455905

[B4] Ghany M. G. Buti M. Lampertico P. Lee H. M. (2023). 2022 AASLD-EASL HBV-HDV Treatment Endpoints Conference Faculty. Guidance on treatment endpoints and study design for clinical trials aiming to achieve cure in chronic hepatitis B and D: Report from the 2022 AASLD-EASL HBV-HDV Treatment Endpoints Conference. Hepatology 78, 1654–1673. doi: 10.1097/HEP.0000000000000431, PMID: 37326326 PMC12373085

[B5] Huang L. Zhang H. Kang X. Chen Z. Wang L. Zeng Y. (2023). Efficacy of pegylated interferon α-2b plus entecavir therapy and predictors of treatment success in children with chronic hepatitis B. Front. Immunol. 14. doi: 10.3389/fimmu.2023.1282922, PMID: 38111577 PMC10726036

[B6] Kennedy P. T. F. Sandalova E. Jo J. Gill U. Ushiro-Lumb I. Tan A. T. . (2012). Preserved T-cell function in children and young adults with immune-tolerant chronic hepatitis B. Gastroenterology. 143, 637–645. doi: 10.1053/j.gastro.2012.06.009, PMID: 22710188

[B7] Kosaka M. Fujino H. Tsuge M. Yamaoka K. Fujii Y. Uchikawa S. . (2025). Usefulness of serum HBV RNA levels for predicting antiviral response to entecavir treatment in patients with chronic hepatitis B. J. Gastroenterol. 60, 469–478. doi: 10.1007/s00535-025-02211-5, PMID: 39841247 PMC11922970

[B8] Lai X. OuYang W. Li S. Qiu J. Zhang H. Jiang T. . (2024). Predictive role of early treatment dynamics of HBV RNA and HBcrAg for HBeAg seroconversion in children with chronic hepatitis B. J. Med. Virol. 96, e29670. doi: 10.1002/jmv.29670, PMID: 38773810

[B9] Le Bert N. Gill U. S. Hong M. Kunasegaran K. Tan D. Z. M. Ahmad R. . (2020). Effects of hepatitis B surface antigen on virus-specific and global T cells in patients with chronic hepatitis B virus infection. Gastroenterology. 159, 652–664. doi: 10.1053/j.gastro.2020.04.019, PMID: 32302614

[B10] Lee S. K. Nam S. W. Jang J. W. Kwon J. H. (2024). Long-term HBsAg titer kinetics with entecavir/tenofovir: implications for predicting functional cure and low levels. Diagnostics (Basel) 14, 495. doi: 10.3390/diagnostics14050495, PMID: 38472967 PMC10931114

[B11] Li J. Fan P. Xu Z. Dong Y. Wang F. Hong W. . (2023). Functional cure of chronic hepatitis B with antiviral treatment in children having high-level viremia and normal or mildly elevated serum aminotransferase. J. Clin. Transl. Hepatol. 11, 1011–1022. doi: 10.14218/JCTH.2023.00014, PMID: 37577220 PMC10412703

[B12] Lim S. G. Phyo W. W. Ling J. Z. J. Cloherty G. Butler E. K. Kuhns M. C. . (2021). Comparative biomarkers for HBsAg loss with antiviral therapy shows dominant influence of quantitative HBsAg (qHBsAg). Aliment Pharmacol. Ther. 53, 172–182. doi: 10.1111/apt.16149, PMID: 33159496

[B13] Mak L. Y. Anderson M. Stec M. Chung M. S. Wong D. K. Hui R. W. . (2025). Longitudinal profile of plasma pregenomic RNA in patients with chronic hepatitis B infection on long-term nucleoside analogues and its interaction with clinical parameters. Clin. Mol. Hepatol. 31, 460–473. doi: 10.3350/cmh.2024.0724, PMID: 39722611 PMC12016600

[B14] Mak L. Y. Cloherty G. Wong D. K. Gersch J. Seto W. K. Fung J. . (2021). HBV RNA profiles in patients with chronic hepatitis B under different disease phases and antiviral therapy. Hepatology. 73, 2167–2179. doi: 10.1002/hep.31616, PMID: 33159329

[B15] Mak L. Y. Hui R. W. Fung J. Seto W. K. Yuen M. F. (2023a). The role of different viral biomarkers on the management of chronic hepatitis B. Clin. Mol. Hepatol. 29, 263–276. doi: 10.3350/cmh.2022.0448, PMID: 36655304 PMC10121282

[B16] Mak L. Y. Wong D. Kuchta A. Hilfiker M. Hamilton A. Chow N. . (2023b). Hepatitis B virus pre-genomic RNA and hepatitis B core-related antigen reductions at week 4 predict favourable hepatitis B surface antigen response upon long-term nucleos(t)ide analogue in chronic hepatitis B. Clin. Mol. Hepatol. 29, 146–162. doi: 10.3350/cmh.2022.0172, PMID: 35989092 PMC9845664

[B17] Martinez M. G. Boyd A. Combe E. Testoni B. Zoulim F. (2021). Covalently closed circular DNA: The ultimate therapeutic target for curing HBV infections. J. Hepatol. 75, 706–717. doi: 10.1016/j.jhep.2021.05.013, PMID: 34051332

[B18] Polaris Observatory Collaborators (2023). Global prevalence, cascade of care, and prophylaxis coverage of hepatitis B in 2022: a modelling study. Lancet Gastroenterol. Hepatol. 8, 879–907. doi: 10.1016/S2468-1253(23)00197-8, PMID: 37517414

[B19] Taddese M. Grudda T. Belluccini G. Anderson M. Cloherty G. Hwang H. S. . (2025). Transcription of hepatitis B surface antigen shifts from cccDNA to integrated HBV DNA during treatment. J. Clin. Invest 135, e184243. doi: 10.1172/JCI184243, PMID: 39898797 PMC11910228

[B20] Terrault N. A. Lok A. S. F. McMahon B. J. Chang K. M. Hwang J. P. Jonas M. M. . (2018). Update on prevention, diagnosis, and treatment of chronic hepatitis B: AASLD 2018 hepatitis B guidance. Hepatology. 67, 1560–1599. doi: 10.1002/hep.29800, PMID: 29405329 PMC5975958

[B21] Tian Y. Yu H. Chen J. (2025). Emerging serum biomarkers for chronic hepatitis B: focus on serum HBV RNA and HBcrAg. J. Clin. Transl. Hepatol. 13, 766–775. doi: 10.14218/JCTH.2025.00064, PMID: 40951536 PMC12422880

[B22] van Bömmel F. Bartens A. Mysickova A. Hofmann J. Krüger D. H. Berg T. . (2015). Serum hepatitis B virus RNA levels as an early predictor of hepatitis B envelope antigen seroconversion during treatment with polymerase inhibitors. Hepatology. 61, 66–76. doi: 10.1002/hep.27381, PMID: 25132147

[B23] Wang F. S. Li J. Zhang C. (2024). Why is the functional cure rate of young children with chronic hepatitis B receiving antiviral therapy considerably high? Hepatol. Int. 18, 296–298. doi: 10.1007/s12072-023-10597-8, PMID: 37907721

[B24] Wang J. Yu Y. Li G. Shen C. Meng Z. Zheng J. . (2017). Relationship between serum HBV-RNA levels and intrahepatic viral as well as histologic activity markers in entecavir-treated patients. J. Hepatol. S0168-8278(17)32261-4. doi: 10.1016/j.jhep.2017.08.021, PMID: 28870671

[B25] World Health Organization (2024). Global hepatitis report 2024: action for access in low- and middle-incomes countries. Available online at: https://ww.who.int/publications/i/item/9789240091672 (Accessed April 9, 2024).

[B26] Wu S. Kang H. Huang E. Wu W. Liu L. Chen R. . (2025). Serum HBV RNA as a predictive biomarker for HBeAg seroconversion during entecavir and tenofovir disoproxil fumarate therapy in chronic hepatitis B patients. Rev. Esp Enferm Dig. 117, 645–653. doi: 10.17235/reed.2025.11226/2025, PMID: 40575888

[B27] Wu Y. Wen J. Tang G. Zhang J. Xin J. (2021). On-treatment HBV RNA dynamic predicts entecavir-induced HBeAg seroconversion in children with chronic hepatitis B. J. Infect. 83, 594–600. doi: 10.1016/j.jinf.2021.08.044, PMID: 34474058

[B28] Wu X. Yao Z. Lai X. Gu Y. Peng S. (2023). Age at treatment initiation predicts response in children with chronic hepatitis B. Aliment Pharmacol. Ther. 58, 866–873. doi: 10.1111/apt.17667, PMID: 37589263

[B29] Xia M. Chi H. Wu Y. Hansen B. E. Li Z. Liu S. . (2021). Serum hepatitis B virus RNA level is associated with biochemical relapse in patients with chronic hepatitis B infection who discontinue nucleos(t)ide analogue treatment. Aliment Pharmacol. Ther. 54, 709–714. doi: 10.1111/apt.16538, PMID: 34275138

[B30] Xia Y. Guo H. (2020). Hepatitis B virus cccDNA: Formation, regulation and therapeutic potential. Antiviral Res. 180, 104824. doi: 10.1016/j.antiviral.2020.104824, PMID: 32450266 PMC7387223

[B31] You H. Wang F. Li T. Xu X. Sun Y. Nan Y. . (2023). Guidelines for the prevention and treatment of chronic hepatitis B (version 2022). J. Clin. Transl. Hepatol. 11, 1425–1442. doi: 10.14218/JCTH.2023.00320, PMID: 37719965 PMC10500285

[B32] Zhang M. Li J. Xu Z. Fan P. Dong Y. Wang F. . (2024). Functional cure is associated with younger age in children undergoing antiviral treatment for active chronic hepatitis B. Hepatol. Int. 18, 435–448. doi: 10.1007/s12072-023-10631-9, PMID: 38376650 PMC11014810

[B33] Zhu S. Dong Y. Wang L. Liu W. Zhao P. (2019). Early initiation of antiviral therapy contributes to a rapid and significant loss of serum HBsAg in infantile-onset hepatitis B. J. Hepatol. 71, 871–875. doi: 10.1016/j.jhep.2019.06.009, PMID: 31228491

