# Supplementary material for: Hepatitis B virus RNA and hepatitis B surface antigen kinetics predict treatment outcomes in children with chronic hepatitis B
Source: Front Cell Infect Microbiol. 2026 Feb 3;16:1746541. doi: 10.3389/fcimb.2026.1746541 (PMC12909504; doi:10.3389/fcimb.2026.1746541)

**Supplementary Figure 1.** **Differential undetectability rates of serum HBV DNA, pgRNA, and HBsAg throughout 96 weeks of NA therapy.** HBV, hepatitis B virus; pgRNA, pregenomic RNA; HBsAg, hepatitis B surface antigen; NA, nucleos(t)ide analogue.


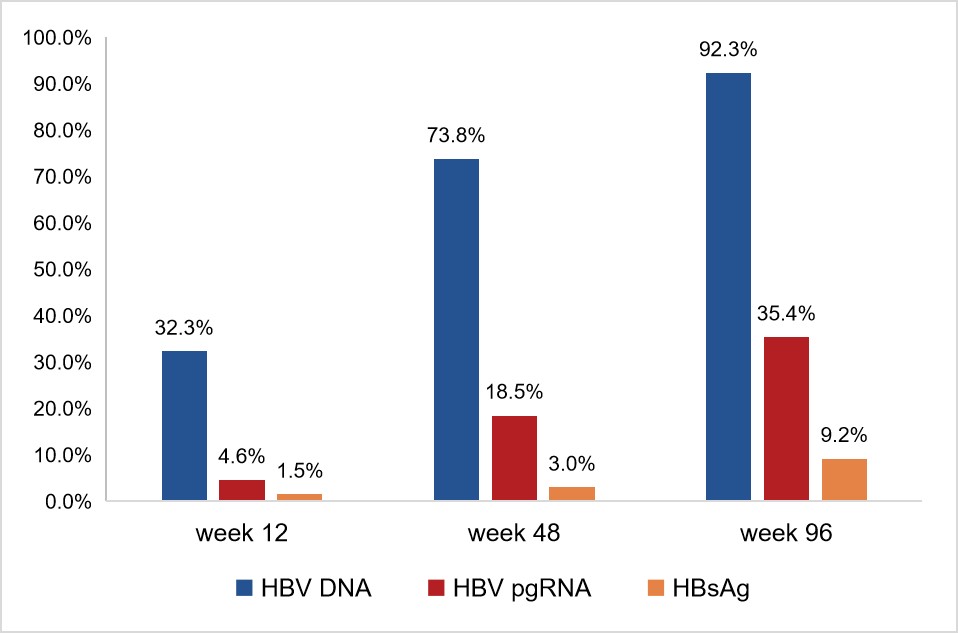

Supplement: Supplementary file 1 [file Supplementaryfile1.docx]
