# Supplementary material for: Hepatitis B virus RNA and hepatitis B surface antigen kinetics predict treatment outcomes in children with chronic hepatitis B
Source: Front Cell Infect Microbiol. 2026 Feb 3;16:1746541. doi: 10.3389/fcimb.2026.1746541 (PMC12909504; doi:10.3389/fcimb.2026.1746541)

**Supplementary Figure 2. C**umulative incidence of HBeAg seroconversion stratified by HBV pgRNA decline at week 12.**** Kaplan-Meier curves show the cumulative incidence of HBeAg seroconversion in patients grouped by the magnitude of pgRNA decline at week 12 (High: ≥1.09 log₁₀ copies/mL; Low: <1.09 log₁₀ copies/mL). Statistical significance was determined by the log-rank test. HBeAg, hepatitis e antigen; pgRNA, pregenomic RNA.


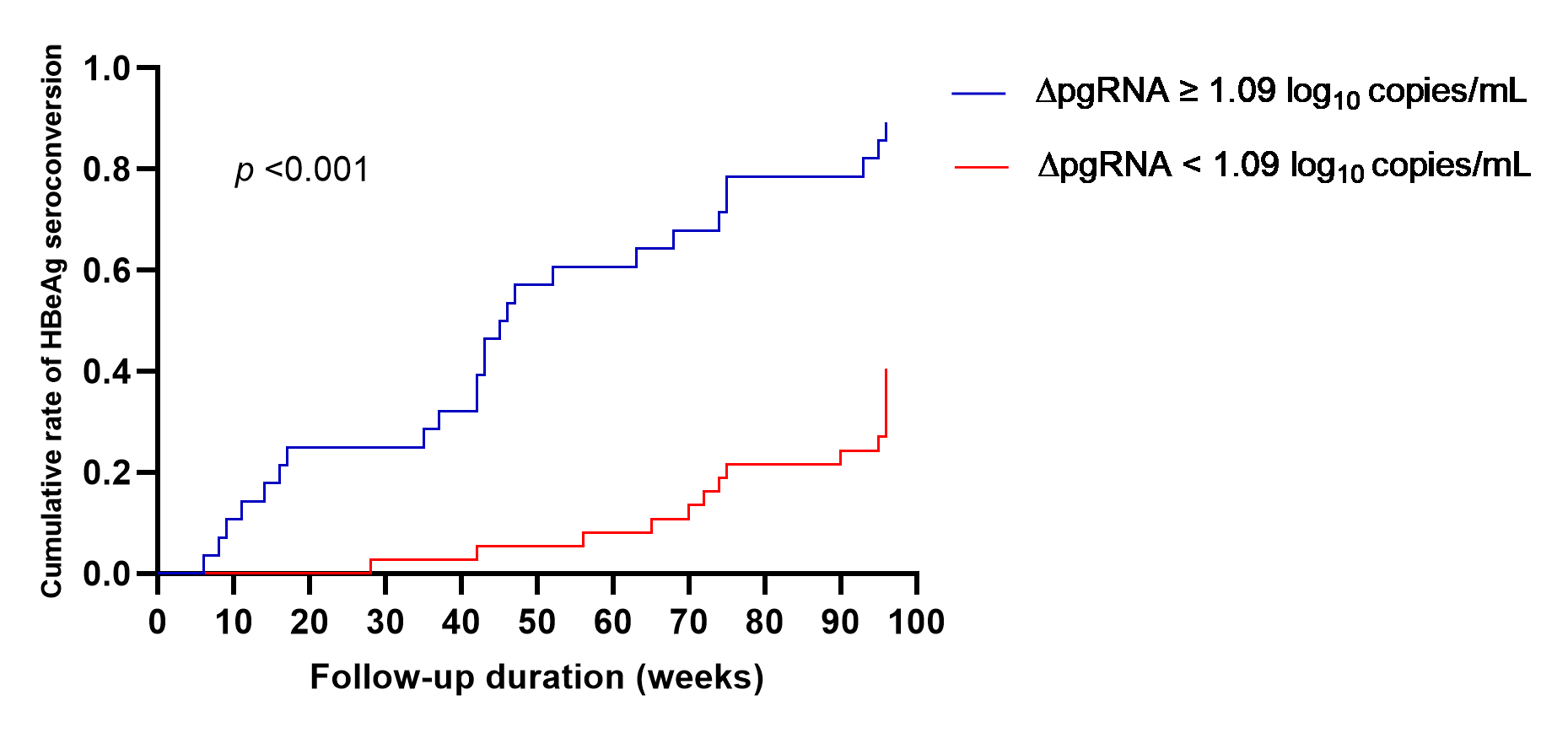

Supplement: Supplementary file 2 [file Supplementaryfile2.docx]
