# Supplementary material for: Hepatitis B virus RNA and hepatitis B surface antigen kinetics predict treatment outcomes in children with chronic hepatitis B
Source: Front Cell Infect Microbiol. 2026 Feb 3;16:1746541. doi: 10.3389/fcimb.2026.1746541 (PMC12909504; doi:10.3389/fcimb.2026.1746541)
Supplement: Supplementary file 3 [file Table1.docx]

**Supplementary Table 1. Correlation coefficients between viral biomarkers during treatment (n=65)**

|  | HBV DNA | HBsAg |
| --- | --- | --- |
| **Week 12** |  |  |
| HBV pgRNA | 0.800 (*p* < 0.001) | 0.661 (*p* < 0.001) |
| HBV DNA | - | 0.660 (*p* < 0.001) |
| **Week 48** |  |  |
| HBV pgRNA | 0.684 (*p* < 0.001) | 0.600 (*p* < 0.001) |
| HBV DNA | - | 0.481 (*p* < 0.001) |
| **Week 96** |  |  |
| HBV pgRNA | 0.474 (*p* < 0.001) | 0.564 (*p* < 0.001) |
| HBV DNA | - | 0.428 (*p* < 0.001) |

Abbreviations: HBV, hepatitis B virus; HBsAg, hepatitis B surface antigen; pgRNA, pregenomic RNA.
