# Supplementary material for: Hepatitis B virus RNA and hepatitis B surface antigen kinetics predict treatment outcomes in children with chronic hepatitis B
Source: Front Cell Infect Microbiol. 2026 Feb 3;16:1746541. doi: 10.3389/fcimb.2026.1746541 (PMC12909504; doi:10.3389/fcimb.2026.1746541)
Supplement: Supplementary file 4 [file Table2.doc]

**Supplementary Table 2.** Median reduction in serum viral markers at different time points during 96 weeks of NA therapy

| **Viral marker** | **HBeAg seroconversion**  **(n=40)** | **Non-HBeAg seroconversion**  **(n=25)** | ***p*-value** | **HBsAg loss**  **(n=20)** | **Non-HBsAg loss**  **(n=45)** | ***p*-value** |
| --- | --- | --- | --- | --- | --- | --- |
| pgRNA, log_10_ copies/mL |  |  |  |  |  |  |
| Week 12 | 1.69(0.50-2.83) | 0.14(-0.26-0.79) | <0.001 | 1.75(-0.01-3.75) | 0.52(-0.06-1.67) | 0.048 |
| Week 48 | 3.93(2.64-5.35) | 0.74(0.28-2.32) | <0.001 | 4.14(2.51-5.49) | 2.33(0.63-3.53) | 0.003 |
| Week 96 | 4.88(3.72-5.64) | 1.60(0.76-2.87) | <0.001 | 4.94(3.92-5.49) | 2.81(1.32-4.52) | 0.003 |
| HBsAg, log_10_ IU/mL |  |  |  |  |  |  |
| Week 12 | 0.59(0.14-1.83) | 0.29(0.08-0.51) | 0.066 | 1.33(0.27-2.14) | 0.29(0.05-0.68) | <0.001 |
| Week 48 | 1.53(0.53-2.61) | 0.38(0.15-0.74) | 0.001 | 2.58(1.65-3.12) | 0.51(0.13-1.05) | <0.001 |
| Week 96 | 1.81(0.66-3.63) | 0.85(0.37-1.36) | 0.005 | 3.58(2.68-5.51) | 0.79(0.34-1.40) | <0.001 |
| HBV DNA, log_10_ IU/mL |  |  |  |  |  |  |
| Week 12 | 4.49(3.67-5.23) | 3.95(3.60-4.65) | 0.143 | 4.35(3.65-5.05) | 4.11(3.65-5.21) | 0.902 |
| Week 48 | 5.21(4.48-5.83) | 4.75(4.06-5.42) | 0.151 | 5.21(4.58-5.83) | 4.96(4.13-5.59) | 0.639 |
| Week 96 | 5.36(4.58-5.86) | 5.39(4.53-5.77) | 0.823 | 5.33(4.58-5.85) | 5.39(4.50-5.77) | 0.935 |

Data are presented as median (interquartile range). Abbreviations: NA, nucleos(t)ide analogue; HBV, hepatitis B virus; HBsAg, hepatitis B surface antigen; pgRNA, pregenomic RNA; HBeAg, hepatitis B e antigen.
