# Supplementary material for: Hepatitis B virus RNA and hepatitis B surface antigen kinetics predict treatment outcomes in children with chronic hepatitis B
Source: Front Cell Infect Microbiol. 2026 Feb 3;16:1746541. doi: 10.3389/fcimb.2026.1746541 (PMC12909504; doi:10.3389/fcimb.2026.1746541)
Supplement: Supplementary file 5 [file Table3.doc]

| **Viral marker** | **HBeAg seroconversion (n=40)** | |  | **Non-HBeAg seroconversion (n=25)** | |  | **Mann-Whitney U Test Results** | | | |
| --- | --- | --- | --- | --- | --- | --- | --- | --- | --- | --- |
|  | **Median ( IQR)** | **Mean rank** |  | **Median ( IQR)** | **Mean rank** |  | **Rank difference** | **U value** | **Z values** | ***p***-value |
| pgRNA, log_10_ copies/mL |  |  |  |  |  |  |  |  |  |  |
| Week 0 | 7.11(6.05-7.76) | 30.08 |  | 7.40(6.92-8.05) | 37.68 |  | 7.60 | 383.00 | -1.578 | 0.115 |
| Week 12 | 4.97(3.55-6.99) | 24.80 |  | 7.31(6.47-7.70) | 46.12 |  | 21.23 | 172.00 | -4.423 | <0.001 |
| Week 48 | 2.71(1.70-3.71) | 22.01 |  | 6.65(5.59-6.98) | 50.58 |  | 28.57 | 60.50 | -5.945 | <0.001 |
| Week 96 | 1.70(1.70-3.05) | 22.81 |  | 5.61(4.23-6.62) | 49.30 |  | 26.49 | 92.50 | -5.621 | <0.001 |
| HBsAg, log_10_ IU/mL |  |  |  |  |  |  |  |  |  |  |
| Week 0 | 4.06(3.67-4.59) | 28.93 |  | 4.48(4.09-4.72) | 39.52 |  | 10.59 | 337.00 | -2.198 | 0.028 |
| Week 12 | 3.31(2.61-3.99) | 26.65 |  | 4.16(3.54-4.48) | 43.16 |  | 16.51 | 246.00 | -3.425 | <0.001 |
| Week 48 | 2.64(1.73-3.46) | 25.03 |  | 3.99(3.33-4.38) | 45.76 |  | 20.73 | 181.00 | -4.302 | <0.001 |
| Week 96 | 2.28(-0.18-3.25) | 25.39 |  | 3.43(3.23-4.23) | 45.18 |  | 19.79 | 195.50 | -4.109 | <0.001 |
| HBV DNA, log_10_ IU/mL |  |  |  |  |  |  |  |  |  |  |
| Week 0 | 7.96(7.18-8.46) | 31.53 |  | 8.16(7.67-8.45) | 35.36 |  | 3.83 | 441.00 | -0.796 | 0.426 |
| Week 12 | 2.67(2.60-3.62) | 26.99 |  | 3.63(3.19-4.57) | 42.62 |  | 15.63 | 259.50 | -3.299 | <0.001 |
| Week 48 | 2.60(2.60-2.60) | 26.79 |  | 3.04(2.60-3.82) | 42.94 |  | 16.15 | 251.50 | -4.335 | <0.001 |
| Week 96 | 2.60(2.60-2.60) | 30.00 |  | 2.60(2.60-2.64) | 37.80 |  | 7.80 | 380.00 | -3.222 | 0.001 |

**Supplementary Table 3. Detailed comparison of virological markers between patients with and without HBeAg seroconversion**

Values below the assay-specific lower limits of detection were assigned the respective LLOD value prior to log₁₀ transformation, as detailed in the Methods. Abbreviations: IQR, interquartile range; HBV, hepatitis B virus; HBsAg, hepatitis B surface antigen; pgRNA, pregenomic RNA; HBeAg, hepatitis B e antigen.
