# Supplementary material for: Hepatitis B virus RNA and hepatitis B surface antigen kinetics predict treatment outcomes in children with chronic hepatitis B
Source: Front Cell Infect Microbiol. 2026 Feb 3;16:1746541. doi: 10.3389/fcimb.2026.1746541 (PMC12909504; doi:10.3389/fcimb.2026.1746541)
Supplement: Supplementary file 6 [file Table4.docx]

| **Viral marker** | **HBsAg loss (n=20)** | |  | **Non-HBsAg loss (n=45)** | |  | **Mann-Whitney U Test Results** | | | |
| --- | --- | --- | --- | --- | --- | --- | --- | --- | --- | --- |
|  | **Median ( IQR)** | **Mean rank** |  | **Median ( IQR)** | **Mean rank** |  | **Rank difference** | **U value** | **Z values** | ***p***-value |
| pgRNA, log10 copies/mL |  |  |  |  |  |  |  |  |  |  |
| Week 0 | 7.13(5.89-7.79) | 30.88 |  | 7.36(6.53-7.77) | 33.94 |  | 3.06 | 407.50 | -0.604 | 0.546 |
| Week 12 | 5.89(3.23-6.99) | 25.25 |  | 6.64(4.97-7.49) | 36.44 |  | 11.19 | 295.00 | -2.203 | 0.028 |
| Week 48 | 2.27(1.70-4.18) | 21.73 |  | 4.86(2.86-6.69) | 38.01 |  | 16.28 | 224.50 | -3.215 | 0.001 |
| Week 96 | 1.70(1.70-2.98) | 20.95 |  | 3.53(1.80-5.93) | 38.36 |  | 17.14 | 209.00 | -3.504 | <0.001 |
| HBsAg, log10 IU/mL |  |  |  |  |  |  |  |  |  |  |
| Week 0 | 4.16(3.62-4.66) | 30.83 |  | 4.24(3.91-4.68) | 33.97 |  | 3.14 | 406.50 | -0.618 | 0.536 |
| Week 12 | 2.73(1.93-3.92) | 21.63 |  | 3.80(3.35-4.40) | 38.06 |  | 16.43 | 222.50 | -3.234 | 0.001 |
| Week 48 | 1.76(0.83-2.26) | 13.40 |  | 3.46(3.31-4.13) | 41.71 |  | 28.31 | 58.00 | -5.573 | <0.001 |
| Week 96 | -0.39(-1.30-1.32) | 10.53 |  | 3.35(3.10-3.97) | 42.99 |  | 32.46 | 0.50 | -6.393 | <0.001 |
| HBV DNA, log10 IU/mL |  |  |  |  |  |  |  |  |  |  |
| Week 0 | 7.93(7.18-8.45) | 31.38 |  | 8.03(7.46-8.46) | 33.72 |  | 2.44 | 417.50 | -0.462 | 0.644 |
| Week 12 | 2.64(2.60-4.06) | 28.33 |  | 3.29(2.64-4.23) | 35.08 |  | 6.75 | 356.50 | -1.352 | 0.176 |
| Week 48 | 2.60(2.60-2.60) | 27.65 |  | 2.60(2.60-3.18) | 35.38 |  | 7.73 | 343.00 | -1.968 | 0.049 |
| Week 96 | 2.60(2.60-2.60) | 30.00 |  | 2.60(2.60-2.60) | 34.33 |  | 4.33 | 390.00 | -1.698 | 0.089 |

**Supplementary Table 4. Detailed comparison of virological markers between patients with and without HBsAg loss**

Values below the assay-specific lower limits of detection were assigned the respective LLOD value prior to log₁₀ transformation, as detailed in the Methods. Abbreviations: IQR, interquartile range; HBV, hepatitis B virus; HBsAg, hepatitis B surface antigen; pgRNA, pregenomic RNA; HBeAg, hepatitis B e antigen.
